# Cross Effects of Diets and Rearing Temperatures on Gastrointestinal Evacuation and Growth Performance in Adult Sabah Groupers (*Epinephelus fuscoguttatus* × *E*. *lanceolatus*)

**DOI:** 10.3390/ani12223172

**Published:** 2022-11-16

**Authors:** Simon Kumar Das, Moumita De, Noorashikin Md Noor, Yosni Bakar, Zaidi Che Cob, Mazlan Abd. Ghaffar

**Affiliations:** 1 Department of Earth Sciences and Environment, Faculty of Science and Technology, Universiti Kebangsaan Malaysia, Bangi 43600, Selangor, Malaysia; 2 Marine Ecosystem Research Center, Faculty of Science and Technology, Universiti Kebangsaan Malaysia, Bangi 43600, Selangor, Malaysia; 3 Earth Observation Centre, Institute of Climate Change, Universiti Kebangsaan Malaysia, Bangi 43600, Selangor, Malaysia; 4 Institute of Marine Biotechnology, University Malaysia Terengganu, Kuala Terengganu 21030, Terengganu, Malaysia

**Keywords:** consumption, digestion, X-radiography, serial slaughter, gastrointestinal evacuation

## Abstract

**Simple Summary:**

The commercially popular Sabah grouper has gained attention due to its high growth rate. Our preliminary study briefly reported how temperature and diet affect gastric emptying time and specific growth rate of hybrid Sabah grouper; however, a comprehensive study on gastrointestinal evacuation GE (Gastrointestinal time together with gastric emptying rate) of adult hybrid Sabah grouper at different temperatures and diets has yet to be conducted. In this follow-up study, we suggest that the optimum temperature and feed for adult Sabah grouper are 30 °C and trash fish, respectively.

**Abstract:**

This study explores the gastrointestinal evacuation time (GET) and gastrointestinal evacuation rate (GER) of the popular Sabah grouper (*Epinephelus fuscoguttatus* × *E*. *lanceolatus*) adults using two established methods (X-radiography and serial slaughter) and square root modeling using different temperatures: 28 °C, 30 °C, 32 °C, and 34 °C and different diets: pellet (ash: 11.4 ± 0.08; moisture: 29.0 ± 0.01; protein 37.5 ± 0.80; lipid 15.0 ± 0.13) and trash fish: *Sardinella* sp. (ash: 2.3 ± 0.15; moisture: 78.5 ± 0.33; protein 55.4 ± 0.62; lipid 7.3 ± 0.25) and the impact on growth indices. The results indicate that the GET shortened as temperature increased from 28 °C to 30 °C; however, it was prolonged when it surged to 32 °C and 34 °C. The groupers fed with trash fish at a temperature of 30 °C had the shortest GER (0.41 ± 0.10 g hr^−1^) whereas groupers fed with pellet at 34 °C had the longest GER (0.95 ± 0.02 g hr^−1^). Likewise, the highest SGR (16.25 ± 2.11% day^−1^) was observed at 30 °C for groupers fed with a trash fish diet. The condition (K) value was lowest at 34 °C for groupers fed with a pellet diet (1.01 ± 0.04) and highest at 30 °C for groupers fed with trash fish (1.45 ± 0.04). Our results suggest that temperature and diet influence growth indices and GE of adult Sabah groupers. Incorporation of this information will allow better management of this commercially important grouper species when reared in a controlled aquaculture environment.

## 1. Introduction

Gastrointestinal evacuation (GE) is defined as product emptying from the stomach to the intestine through the pyloric sphincter, and the rhythm or rate of its occurrence as measured in weight (g) or proportion (%) per unit of time (t) [1]. Feeding rhythm and time for GE can be influenced by temperature, consumption, diet quality, activity, body size, intestinal capacity, satiety, and metabolic rhythm [2]. Fish, which are poikilothermic, exhibit increased metabolic rates, consumption, and evacuation rates with increasing water temperature up to a maximal thermal threshold [1]. Water temperature also influences the GE process of fish. Besides temperature, GE is also influenced by the following factors: size of food particles (the bigger the food particles the lower the GE), salinity (as salinity increases GE decreases), frequency of consumption (the higher the frequency of consumption the lower the GE), and dietary components (GE is faster in feed with fiber) [2,3,4,5,6].

GE in fish has drawn research interest over recent decades, especially in the aquaculture industry [7,8,9]. Several techniques have been developed to investigate GE in fish. These techniques include serial slaughter and X-radiography [8,10]. In serial slaughter, experimental fish are given a meal of a known weight or volume before the residual of food extracted from the digestive tract is then measured by serial slaughter at successive intervals after feeding. X-radiography is performed by mixing radio-opaque barium sulphate (BaSO_4_) at concentrations of 20–25% with the experimental food.

GER is calculated by fitting data into equations of the gastric emptying curve, namely, exponential, square root, and linear equations [11,12]. A better fit of data is obtained in square root equations if the fish are predatory, whereas the exponential function provides a better fit if the food items consumed by the fish are small and have a low-energy content. Conversely, GER data are best fitted to a linear expression when the meal is hard to digest [13].

Temperature can influence the growth indices (SGR and K) of fish by influencing their feeding rate [14]. This has been reported in marine fishes such as cod *Gadus morhua* L. and turbot *Scophthalmus maximus* L. [15,16]. An optimum temperature is needed for the fish to maximize its growth performance [17]. In addition, the effects of temperature on diets have been revealed in the sea bream *Sparus aurata* and blood snapper *Lutjanus malabricus* [5,18]. Apart from the marine fish, the hybrid Sabah grouper (*Epinephelus fuscoguttatus* × *E*. *lanceolatus*) has gained attention because it grows fast and tastes good. The Sabah grouper is the focus of this paper. Hybridization strategies for Sabah grouper aim to introduce the rapid growth rate of the Giant grouper (*E*. *lanceolatus*) to other species. *Epinephelus lanceolatus* is challenging to breed and raise but introducing its superior genetic features into a hybrid allows for a faster growth rate in the hybrids [4]. The Sabah grouper is developed through the artificial combination of the Giant grouper milt and Tiger grouper eggs. This hybrid has a rapid growth rate, a high survival rate, and a simple larvae cultivation technique, which result in it attracting the attention of aquaculturists.

A preliminary study by De et al. [2] reported that temperature (22 °C, 26 °C, 30 °C, and 34 °C) and diet (pellet and shrimp) influence gastric emptying time and SGR of the juvenile hybrid Sabah grouper. In their study, De et al. [2] used a temperature interval of 4 °C; however, using a minimum temperature of 22 °C was inappropriate as tropical countries never experience such low temperatures. Moreover, using a shrimp diet is expensive and not economically viable for highly carnivorous fish such as the grouper. Additionally, temperature intervals of 4 °C may be inappropriate as temperature changes of 1 °C–2 °C may be biologically important. To address these shortcomings, we conducted this comprehensive study on GET and growth performance of adult hybrid Sabah grouper using the temperature intervals of 2 °C and a temperature range of 28 °C to 34 °C. The current practice by aquaculturists is to raise hybrid grouper fish to advanced larval stage indoors usually at 28 °C. Thereafter the fish are transported to the sea cages where the temperature is higher (>28 °C) and more variable. To overcome the feeding costs associated with a shrimp diet, we substituted shrimp with trash fish. Thus, the goal of this study was twofold: (i) to characterize the gastrointestinal evacuation time and gastrointestinal evacuation rate through X-radiography, serial slaughter, and square root modeling, and (ii) to determine the effects of temperature and diet on the growth indices of adult hybrid Sabah grouper.

## 2. Materials and Methods

### 2.1. Fish Experimental Tank Setup

**Figure 1:**
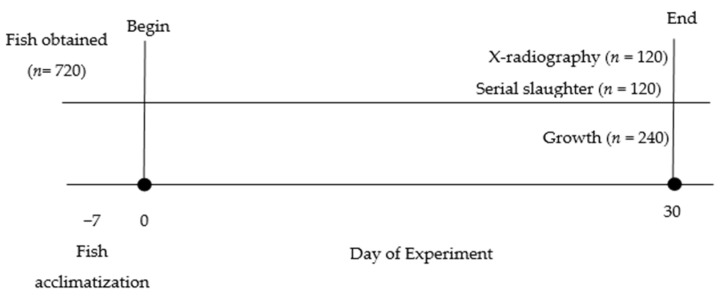
Experiment timeline, where groupers were obtained and acclimatized for 7 days (Day 7), before being distributed to the experimental tank on Day 0. Groupers were distributed evenly among four treatment groups (28, 30, 32, and 34 °C) and among 3 tanks within each group, for a total of 12 tanks. Groupers were cultured for 30 days before growth was measured. Overall, 5 groupers from each tank were removed for X-radiography (5 fish × 3 replicates × 4 temperatures × 2 diets = 120) and serial slaughter experiment (5 fish × 3 replicates × 4 temperatures × 2 diets = 120) while 10 groupers (10 fish × 3 replicates × 4 temperatures × 2 diets = 240) were obtained from each tank for the growth study.

Adult hybrid Sabah groupers (*n* = 730, mean weight = 380.2 ± 1.5 g, mean length = 30.0 ± 0.5 cm) were obtained from the Selangor fish farm (3°58′0″ N, 103°20′0″ E), Malaysia. The fish were brought to the Universiti Kebangsaan Malaysia’s Ocean Science Laboratory and distributed to three stocking tanks (each measuring 2.05 m × 1.33 m × 0.72 m, and with a capacity of 1500 L). Hybrid Sabah groupers were acclimated for seven days (Figure 1) at 28 °C and fed with a pellet diet (HZ Gentian, Johor, Malaysia) as used on the grouper’s farm. After acclimatization, the samples were distributed to 24 experimental tanks. Half of the experimental tanks were labeled and fed with pellet diet (360 fish: 30 fish × 12 tanks), and the remaining 12 tanks were fed with freshly thawed trash fish (*Sardinella* sp.) (360 fish: 30 fish × 12 tanks). Triplicate tanks were used for each of the four temperatures used (28 °C, 30 °C, 32 °C, and 34 °C). Temperature was adjusted daily by 1 °C per day until it reached the desired experimental temperature. Temperature changes were made using chillers (TECO, Milan, Italy) and heaters (EHEIM GmbH, Hamburg, Germany). Once the required temperature was achieved, each experimental aquarium was maintained at that temperature ± 0.5 °C with supplemental aeration and skimming. Hybrid Sabah groupers were fed with pre-weighed commercial pellet diet or freshly thawed chopped trash fish once a day until the fish stopped feeding actively [2]. The point of satiation was identified once fish quit vigorously feeding and food accumulated at the bottom of the tanks over 2 min [8]. The leftover feed was siphoned and weighed to determine the total consumption. Farmers in grouper aquaculture cages have been using floating pellets, which are ideal for satiation feeding, to feed the fish to fullness. Satiation feeding is recommended due to its flexibility and simplicity. With satiation feeding, fish are fed in small batches progressively and constantly until they have enough to eat. We know they have had enough to eat when their rate of feeding lowers prior to stopping entirely. With floating pellets, even a novice fish farmer can conveniently keep track of the eating behavior of fish, feeding them when they are hungry and stopping when they are not. Thus, both underfeeding and overfeeding are prevented concurrently, enabling optimal growth and minimum size variation in each cohort.

The total duration of the experiments was 30 days (d) to keep the increases in relative length and weight at a similar magnitude in all the experiments. Survival of the experimental fish was monitored over the entire rearing period. Fish maintenance, handling, and sampling were conducted according to the protocol and ethics approved by Universiti Kebangsaan Malaysia (FST/2016/SIMON/27-JULY/763-JULY-2016-MAY-2017).

Water temperature, pH, salinity, and dissolved oxygen were checked daily using a water quality meter (HI98107; Hanna Instrumentals, Woonsocket, RI, USA). Total ammonium levels were measured every two days using an NH_3_/NH_4_^+^ test kit (Merck, Darmstadt, Germany). Approximately 20% of the water was replaced daily with filtered clean seawater as feces and debris were siphoned from the tank.

### 2.2. Nutrient Composition of Diets

The nutrient composition of the pellet and trash fish was determined by proximate analysis (Table 1) following the method accredited by the Association of Official Agricultural Chemists. The grounded sample of diets was dried in an oven at 105 °C for 24 h before the moisture loss was calculated [8]. As for ash, the prepared diet samples were placed in a desiccator for cooling before being placed in a muffle furnace at 550 °C for 6 h. Crude protein of dried samples was analyzed using the Kjeldahl method, where sulphuric acid (H_2_SO_4_) was added to the Kjeldahl flask until the solution became clear. For lipid analysis, the dried samples were fixed to the fat extracting apparatus with the sample tube and heated for 4–5 h with a at 60 °C. The samples were then transferred into a vacuum oven at 80 °C. Beakers were then weighed again for lipid percentage. The following formulas were used to calculate the mentioned percentages:

Moisture % = (Sample Fresh weight with crucible − Crucible weight) − (Sample dry weight with crucible − Crucible weight)/Sample fresh weight × 100

Ash % (dry matter basis) = (sample ash weight/sample dry weight) × 100

Crude protein % = (burette reading × Normality of H_2_SO_4_ × 8.75)/(Weight of sample × dry matter %)

Lipid % = (weight of fat/weight of dry sample × dry matter) × 100

### 2.3. Gastrointestinal Evacuation Study

#### 2.3.1. Estimation of Stomach Volume 

The stomach volumes of adult grouper samples were analyzed according to Mazumder et al. [19]. Briefly, each fish’s digestive tract was delicately detached by pulling out the body cavity. A cotton string was strung across the whole anterior of the esophagus to a burette and the posterior of the esophagus to the pyloric sphincter to obtain stomach contents. The total volume of water needed to expand the stomach till it burst was determined as the maximum stomach distension (maximum stomach volume).

#### 2.3.2. Estimation of Maximum Feed Intake

Modeling of satiation feeding was performed to estimate the maximum intake of food by different sizes of fish, since the fish did not eat the same amount of food given. Satiation amount (S_max_) in relation to fish weight was calculated as:

S_max_ = aW^b^

where S_max_ is the maximum feed intake (g), and W is the total weight of the fish (g). The non-linear regression was fitted using Minitab Ver. 17 (StatSoftInc., Tulsa, OK, USA) where a and b were obtained.

#### 2.3.3. GET and GER Calculation 

For determination of GET and GER, X-radiography, serial slaughter, and square root modeling were used. For X-radiography, five individual hybrid Sabah groupers from each group were used. Hybrid Sabah groupers were fed with BasSO_4_ diets. For each 5 g of food (pellet/trash fish), 1 g of BasSO_4_ was added. Upon mixing with BaSO_4_ the pellet diet was dried at 50 °C while the trash fish diet was kept frozen at −15 °C [3]. The fish were starved for two days prior to feeding them BaSO_4_ diets. The hybrid Sabah groupers were anesthetized with Transmore (Nika, Penang, Malaysia) by diluting 0.20 mL of Transmore with 1 L of seawater, and each sample was X-rayed in an X-ray unit (M60, Tokyo, Japan) at 1, 6, 8, 10, 12, 14, 15, 16, and 17 h after feeding. X-ray images were obtained to track the food as it passes along the gut. After being X-rayed, fish were fully recovered in highly aerated seawater. Anesthetized groupers were cautiously treated by putting them on wet cloth and applying STRESS COAT^®^ liquid (API^TM^ Aquarium Pharmaceutical, McLean, VA, USA) to reduce the stress.

For the serial slaughter procedure, five individual hybrid Sabah groupers were fasted for two days and fed without a BaSO_4_ diet (pellet/trash fish). The fish samples were then anesthetized and slaughtered at the same time (0, 1, 6, 8, 10, 12, 14, 15, 16, and 17 h after feeding) intervals as those used for X-ray analysis. Each fish was dissected before the weight of gastrointestinal digesta (g) was recorded. The stomach content at time zero (S_0_) and total stomach content at time t (S_t_) after ingestion of a meal were weighed to the nearest 0.001 g using an electronic balance (Kern Elctronic Scales, Penang, Malaysia).

The serial slaughter data were then analyzed for the GER estimation using the square root mode (Equation (1)) as recommended for other carnivorous marine fishes [3,19].

(1) $$S_{t}=S_{0}(1-S_{0}{}^{\left(\alpha -1\right)}\rho \;\left(1-\alpha \right)\;t{)}^{{\left(1-\alpha \right)}^{-1}})+\xi$$

where S_t_ is the total stomach content at time t after ingestion of a meal of size S_0_, ξ is the random error, α is gastric emptying coefficient (α = 0.5), and *ρ* is the rate constant.

Model parameters for the maximum satiation meal (when S_0_ = maximum satiation amount S_max_), the rate constant *ρ*, and GER were estimated by the nonlinear iterative method using the Levenberg–Marquardt algorithm in Microcal Origin ^TM^ Version 8.0 (OriginLab, Northampton, MA, USA). The nonlinear procedure was first used to examine the starting specifications of the parameters and to evaluate the chi-square (χ^2^) value at each combination of values to determine the optimal set of values that would start the iterative algorithm.

### 2.4. Growth Indices Analysis

Ten groupers from each tank were mildly anesthetized for 10 min with α-methyl quinoline (0.20 for 1 L of sea water) to calculate the total length and weight. The total length (TL) of the fish was measured from the anterior tip of the longest jaw to the most posterior part of the caudal fin using a digital caliper (0.01 cm accuracy). The body weights were measured using an electronic balance (CJ-15 K, Shinko Desnhi, Tokyo, Japan). The growth indices including specific growth rate (SGR), condition factor (K), feeding rate, and feed conversion ratio (FCR), and daily weight gain were calculated from the formulas [20,21]:

SGR (% day^−1^) = 100 × (ln final mass−ln initial mass × day^−1^)

K = 100 WL^−3^

where W is the body weight (g) of each fish and L is TL (cm).

Feeding rate (%/d) = 100 × Total feed consumed/[(Final fish weight + Initial fish weight) × number of fish × number of days/2]

FCR = dry feed consumed (g)/wet weight gain (g)

Daily weight gain (g/day) = Final weight- Initial weight/number of days

### 2.5. Statistical Analysis

Growth indices were analyzed for normality and equality of variances using Kolmogorov–Smirnov tests before two-way ANOVA test. A two-factor factorial model was used to analyze the effects of diet and temperature on the growth indices. Student–Newman–Keuls multiple comparison tests were run to test for significant differences among experimental groups. Statistical analyses were carried out using Minitab Ver. 17 (StatSoftInc., Tulsa, OK, USA).

## 3. Results 

### 3.1. Gastrointestinal Evacuation

**Figure 2:**
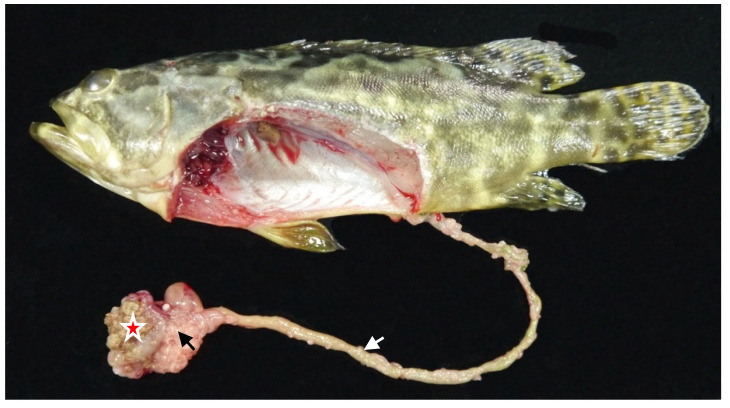
Elementary tract of hybrid Sabah grouper. Star denotes a stomach filled with feed, black arrow indicates pyloric caeca, and white arrow indicate intestine.

**Figure 3:**
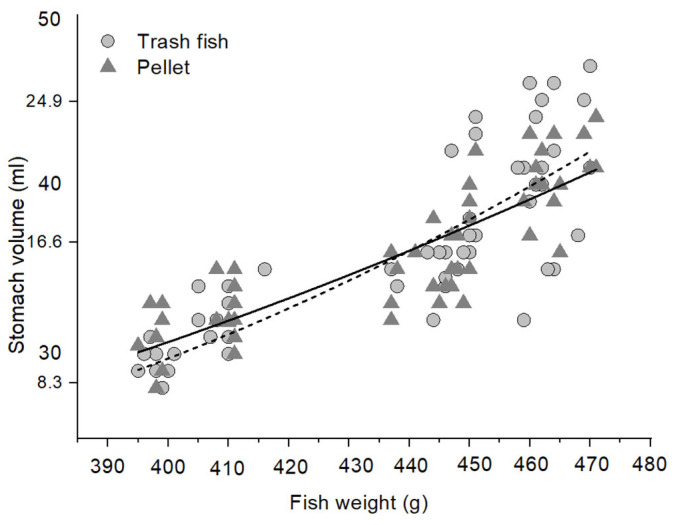
Relationship between stomach volume (mL) and fish weight (g, wet weight) of the adult hybrid Sabah grouper fed, fitted with the allometric model both in pellet and trash fish diets. For the pellet diet, the equation of stomach volume was 0.001 W^1.724^ (chi-square = 4.698; r^2^ = 0.72). Meanwhile, for the trash fish diet, the equation of stomach volume was 0.0002 W^2.12^ (chi-square = 3.424; r^2^ = 0.84).

**Figure 4:**
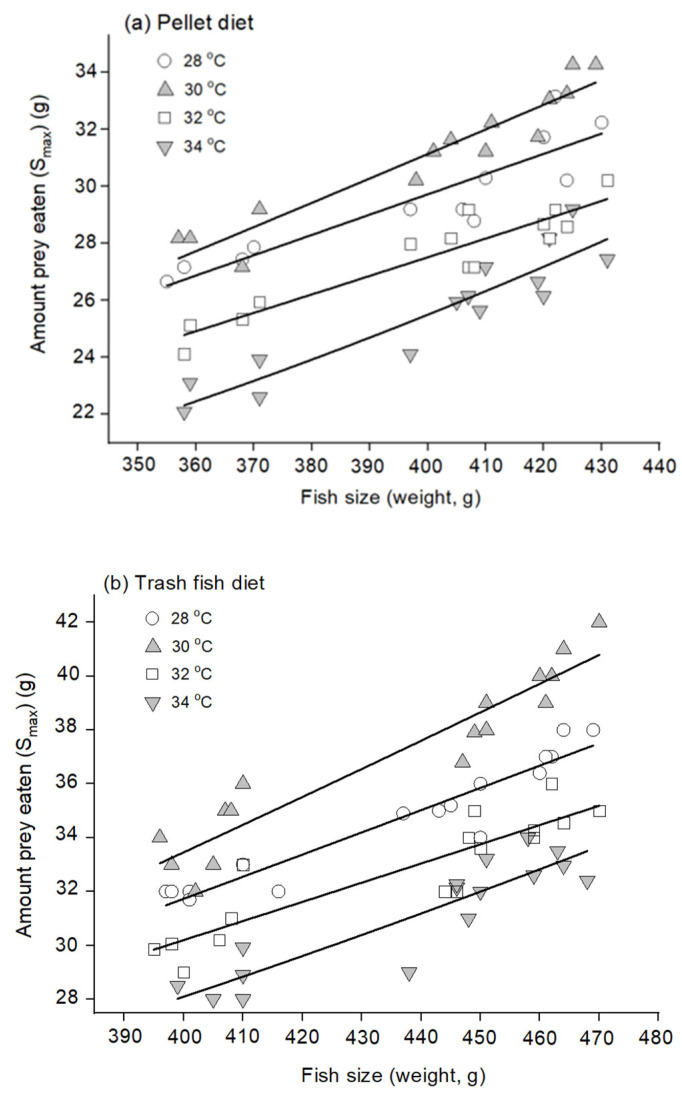
Relationship between the amount of satiation meal (S_max_, g wet weight) and size of fish (W, g) of the adult hybrid Sabah grouper fed with (**a**) pellet, and (**b**) trash fish diet at different temperatures. Allometric model was fitted for all treatments. For the pellet diet, the equation of S_max_ was 0.05 W^0.98^ (r^2^ = 0.82) in 28 °C, 0.03 W^1.08^ (r^2^ = 0.90) in 30 °C, 0.02 W^1.08^ (r^2^ = 0.86) in 32 °C and 0.001 W^1.63^ (r^2^ = 0.85) in 34 °C, respectively. Meanwhile, for the trash fish diet, the equation of S_max_ was 0.06 W^1.04^ (r^2^ = 0.91) in 28 °C, 0.04 W^1.14^ (r^2^ = 0.89) in 30 °C, 0.05 W^1.04^ (r^2^ = 0.80) in 32 °C and 0.008 W^1.37^ (r^2^ = 0.81) in 34 °C, respectively.

The hybrid Sabah grouper stomach was relatively thick-walled with a long intestine, consistent with that of animals with carnivorous feeding habits (Figure 2). The stomach volume of the hybrid Sabah grouper was related with the grouper’s size (W, g), in that the maximum stomach distention was optimally presented by an allometric model for both fish fed with both pellet (volume = 0.0002 W^2.12^) and trash fish (volume = 0.0001 W^1.724^) diets (Figure 3). Subsequently, a nonlinear allometric model was used for all temperatures at which fish were reared regardless of type of diet used. The best fit was observed at 30 °C for both pellet and trash fish diets (r^2^ = 0.90, 0.89; *p* < 0.01, respectively) (Figure 4).

**Figure 5:**
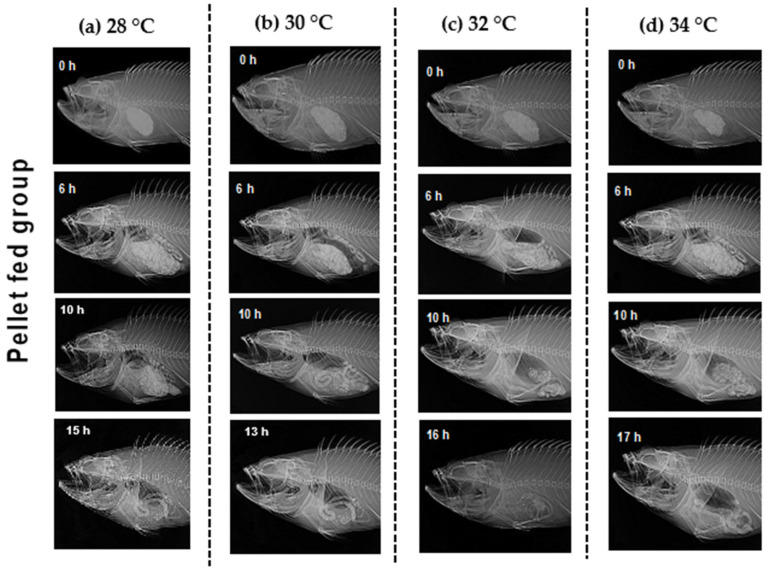
Movement of radio-opaque barium sulphate (BaSO_4_) along the alimentary tract of the adult hybrid Sabah grouper. Each panel shows the passage of ingested BaSO_4_-treated pelleted food through the alimentary tract of the hybrid Sabah group at (**a**–**d**). Five fish were used for each sampling point and temperature. Only X-ray images with notable movement along the tract are shown.

**Figure 6:**
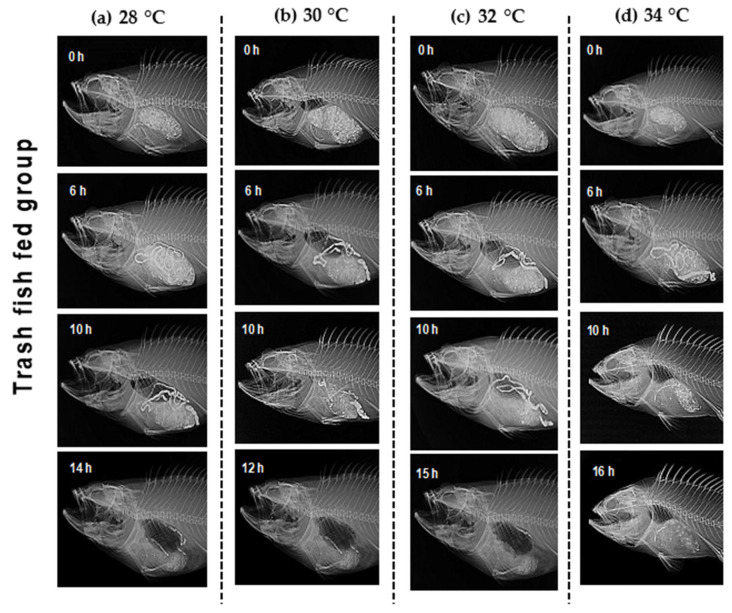
Movement of radio-opaque barium sulphate (BaSO_4_) along the alimentary tract of adult hybrid Sabah grouper. Each panel shows the passage of ingested BaSO_4_-treated trash fish food through the alimentary tract of the hybrid Sabah group at (**a**–**d**). Five fish were used for each sampling point and temperature. Only images with remarkable movement are shown.

X-radiography observations (Figure 5 and Figure 6) indicate the food route in the alimentary tract. GET was shortened when the temperature rose from 28 to 30 °C. However, GET increased when the temperature rose beyond 30 °C to 34 °C. GET was quicker in hybrid Sabah groupers fed with trash fish than in pellet diets at all temperatures. X-ray images showed that the food had completely left the digestive tract (tubular structure consist of four parts: oral cavity; initial region with an esophagus; stomach and pylorus; a medium portion of longer length comprises of pyloric blind; and a terminal region that ends with the anus) after 15 h (28 °C), 13 h (30 °C), 16 h (32 °C), and 17 h (34 °C) after the pellet diet was given (Figure 5, Supplementary Materials Figure S1), as well as after 14 h (28 °C), 12 h (30 °C), 15 h (32 °C), and 16 h (34 °C) after the trash fish diet was fed (Figure 5, Supplementary Materials Figure S2). The shortest GET was marked in the hybrid Sabah grouper cultured at 30 °C and fed with trash fish (Figure 6).

**Figure 7:**
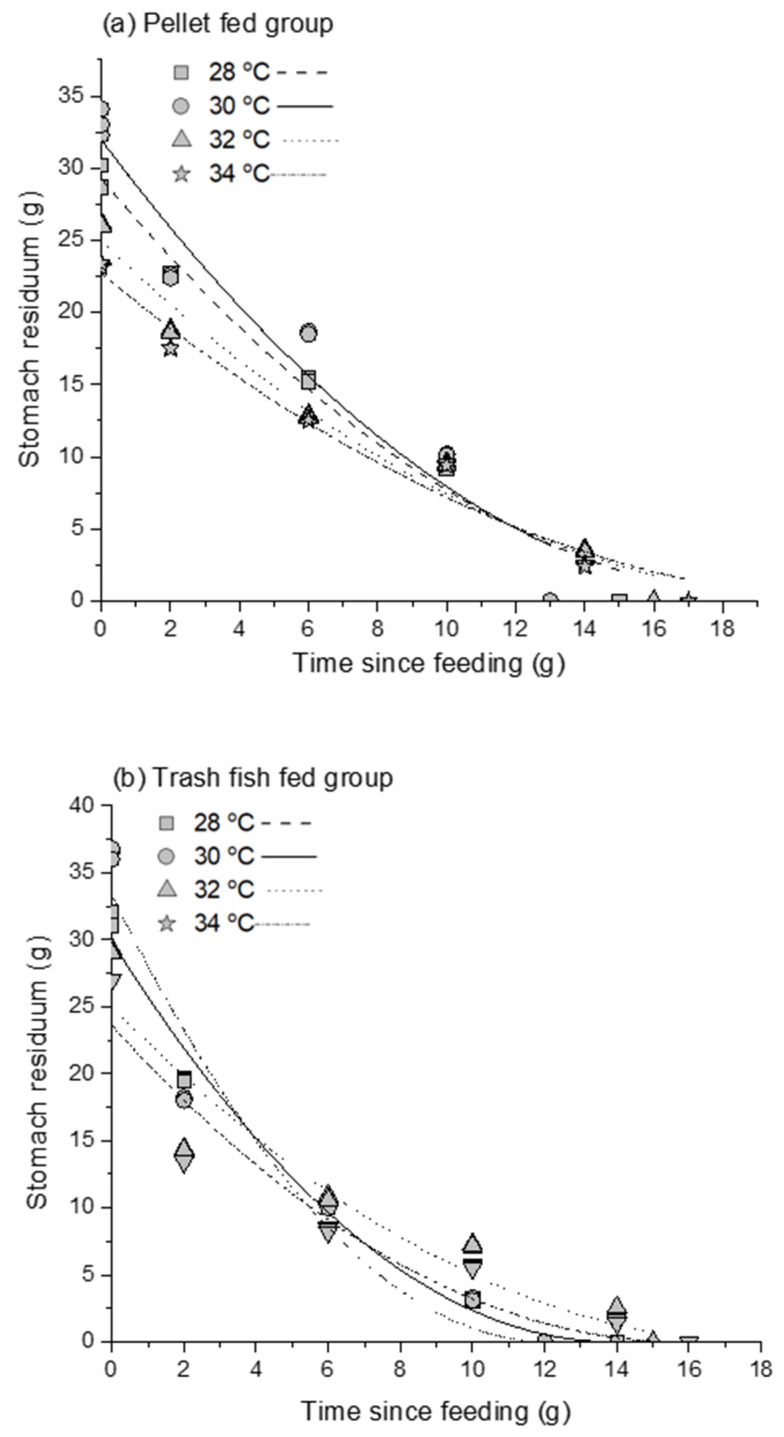
Gastric emptying curve of the adult hybrid Sabah grouper fed with pellet (**a**) and trash fish (**b**) at four different temperatures (28, 30, 32, and 34 °C) fitted through a modified square root model [S_t_ = S_0_ (1−S_0_^(*α*−1)^ *ρ* ^(1−α)^ t)^(1−α)−1^ + ζ], as St is the total stomach content at time t after ingestion of a meal of size S_0_, ξ is the random error, α is gastric emptying coefficient (α = 0.5), and *ρ* is the rate constant.

A modified square root model fit the stomach residuum (S_t_) data at the stated time (t) after feeding with nonlinear regression. The estimated GER values (*ρ*) are presented in Figure 7 and Table 2. The average meal sizes at time zero (S_0_) for the hybrid Sabah grouper used in the study differed at varied temperatures and diet treatments (Table 2). S_0_ was highest when the fish were fed with the trash fish diet at 30 °C and the lowest with the pellet diet at 34 °C. The *ρ* was highest when hybrid Sabah groupers were fed with trash fish at 30 °C and the lowest when hybrid Sabah groupers were fed with pellets at 34 °C. The gastric emptying coefficient (α) used in this model was 0.05 [8]. The model’s regression coefficient (r^2^) ranged between 0.93 and 0.98 for the fish fed with pellet and between 0.90 and 0.98 for the fish fed with trash fish; indicating a good fit of the model to the data in both cases.

### 3.2. Growth Indices

**Figure 8:**
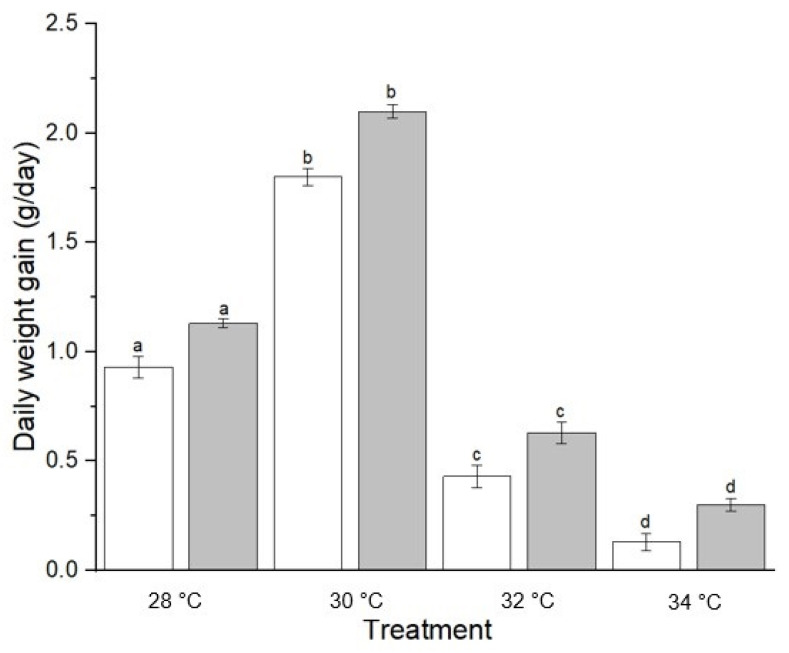
Daily weight gain of hybrid Sabah grouper in treatments at different temperatures and fed for 30 days. White bar represented pellet while gray bar represented trash fish. Different letters indicated significant differences among treatments (*p* < 0.05).

The interaction between diet and temperature was significant for all growth indices but considered as unimportant because the interaction plot, though not quite parallel, was close. Pairwise comparisons of means are presented in Table 3. The main effects of temperature were significant for all growth indices while diet was significant for SGR and K. As the temperature increased from 28 °C to 30 °C, the SGR increased (Table 3). Hybrid Sabah groupers cultured at 30 °C and fed with trash fish showed the highest daily weight gain (*p* < 0.05) (Figure 8). Hybrid Sabah groupers at 34 °C and fed with pellets showed the lowest daily weight gain after the end of the experimental period. Similar trends were observed on the K values. K values were lowest at 34 °C and highest at 30 °C regardless of diet. Further, SGR increased significantly when the temperature increased from 28 °C to 30 °C. However, SGR lowered when the temperature rose from 32 °C to 34 °C. The highest SGR was recorded in hybrid Sabah groupers cultured at 30 °C and fed with trash fish whereas the lowest SGR was recorded at 34 °C in the group fed with pellets. Overall, the highest mean feeding rate was observed among fish at 30 °C, whereas the lowest was in fish at 34 °C. FCR data supported the highest growth performance in fish raised at 30 °C and fed with trash fish. In fact, the highest FCR was observed in 34 °C fish fed with pellets.

## 4. Discussion

A positive correlation between the estimated volume of the stomach and the amount of voluntary food intake by an individual fish has been previously reported [21,22]. However, few such relationships have been derived from direct measurements for stomach volume estimation, regardless of the feeding temperature and diet effect. Stomach volume estimates the maximum stomach capacity, but this parameter seems to underestimate the satiety amounts for the adult hybrid Sabah grouper in the current study at all temperatures and diet types used. Based on the model of the maximum stomach capacity suggested by Mazlan et al. [23], the maximum stomach satiety is two-thirds of the stomach capacity; however, our findings show that the maximum stomach satiety is higher than suggested for both the pellet (0.98–1.63) and trash fish diets (1.04–1.37) at all temperatures used. From this, we concluded that the hybrid Sabah grouper stomach could consume more food at all temperatures than the suggested value, which could be attributed to the high plasticity of the stomach expected of a carnivore like it.

Similarly, Pirhonen et al. [24] reported that the stomach volume is directly proportional to the weight of fish. A previous study noted that the number of satiations decreases as the bodyweight decreases, suggesting that smaller fish eat relatively higher amounts of food than larger fish [25]. In all cases, the hybrid Sabah groupers consumed more trash fish than pelleted feed. The pellet feed is compact and has an artificial flavor, which may induce reluctance to eat, whereas trash fishes are natural and soft and therefore more appealing. The hybrid Sabah groupers ate more at 28 °C and 30 °C, than at 32 °C and 34 °C, indicating that the 28 °C–30 °C temperature range is favorable for these fish. 

Both X-radiography and serial slaughter techniques displayed similar GE patterns, confirming the model’s accuracy. BaSO_4_ has been previously used as an inert marker for fish and does not appear to affect digestion [3]. In this study, a faster GE was recorded in hybrid Sabah groupers fed with trash fish. The faster GE is probably attributed to the effect of type of feed toward the metabolic rate in the fish [26]. The shorter GE further increases the amount of food consumed and also increases the growth rate in the hybrid Sabah grouper. The GET recorded in this study (12–17 h) was shorter than that recorded in previous studies in carnivorous fish such as the whiting *Merlangius merlangus* and the banded archerfish *Toxotes jaculatrix,* which took 70–96 h to complete their stomach evacuation [3,23]. These differences in GET are most likely due to differences in the type of diet used.

A relationship between GE and the food consumed has been documented in the Mahseer *Tor tambroides* and spotted scat *Scatophagus argus* [17,27]. However, GE is not likely to be decreased within the normal range of temperatures in certain species as the gastric acid and enzymes secreted differ at certain temperatures and types of diet [1]. This study has shown the impact of temperature and type of diet on the GE of hybrid Sabah groupers, similarly to that recorded in the brook trout *Salvelinus fontinalis*, and trout *Oncorhynchus mykiss* [28,29]. GET is important in the management of fisheries as the rate of digestion of feeds influences the growth and production rates of the fish. A square root model, similar to those used for other carnivorous fish [3,19] and the GE coefficient, *α*, of 0.5 used in other carnivorous fish [3,19,20] were used to fit GER in this study. A high rate of *GE* increases the digestion process and may increase the nutrient absorption, which is linked to the better growth performance of adult hybrid Sabah groupers.

The growth indices were highest in this study when hybrid Sabah grouper were fed with trash fish and reared at 30 °C. It was observed that hybrid Sabah grouper consumed more food when fed with trash fish than when fed with pellets, and the highest consumption was observed at 30 °C. The better growth parameters of the fish fed with trash fish diet in this study correlates with the higher protein concentration of the trash fish diets than the pellet diet. Similarly, a higher temperature led to higher growth rate in the Atlantic salmon (*S*. *salar*) [30]. Thus, 30 °C seems to be the optimum physiological temperature for adult hybrid Sabah groupers as in their parental species *E. fuscoguttatus* [31]. It was observed that even large fish at 34 °C did not have a high feeding rate. It is suggested that extreme conditions such as excessive warming can decrease the growth rate by lowering the appetite of fish reducing assimilation efficiency (increasing FCR), and shifting energy balance due to the requirement for increased surfacing activity for air breathing [32]. Interestingly, the growth rate of fish under a higher temperature (34 °C) was significantly lower and associated with a higher FCR than that at lower temperatures (28 °C, 30 °C, and 32 °C) as the fish required more food to gain a unit of their body weight.

The optimum growth temperature supported the GE data, which was previously discussed. A condition factor of greater than one showed the wellbeing of fishes fed with different feeds and temperatures. The values of K fed with trash fish and 30 °C were the highest suggesting that the fish group were much more robust than the other treatments. In addition, compared to trash fish, commercial pellets dissolve faster in the stomach resulting in the fish losing control over the evacuation process and the intestines becoming overwhelmed [11]. Diet composition is a significant determinant since fish fed with diverse diets regulate their food consumption rate to keep a relatively constant energy intake while raising the GER of low-energy feeds [13]. The impact of the energy density of trash fish (protein: lipid) on GER across several fish species has been studied and incorporated into general equilibrium models, which have explained their use in determining food intake rates and feeding patterns. Furthermore, fish at 34 °C exhibited a poorer condition irrespective of diet differences, whereas fish reared at 30 °C showed the highest K suggesting that they had a better condition. These findings suggest that elevated sea temperatures of more than 30 °C are expected to affect grouper populations. Similarly, a decrease in K and growth rate was previously reported in the marine reef fish Spiny chromis (*Acanthochromis polyacanthus*) at 31 °C, regardless of the type of food [33]. This further suggests that higher metabolic costs associated with elevated water temperatures, tied with poor foraging ability or diet quality, could decrease the growth rate and K. 

The use of trash fish as the primary feed source is unsustainable and environmentally disruptive. However, due to the greater economic effectiveness of trash fish compared to pellets, farmers continue to frequently favor its use. On the other hand, there seems to be a cause for concern regarding the quantity of raw fish used by the reduction sector and the growing usage of reduced products in aquaculture. Pellet feed has no obvious environmental, cost–benefit, or resource use advantages over trash fish. Pellet feed is solely used because of the advantages it provides in terms of storage capacity and not requiring daily preparation processes prior to feeding, among other things. In light of globalization, it would be improper to restrict the use of trash fish as feed as it is economically viable.

Based on our findings, we predict that global warming will cause a shift in the distribution limits for fish species with a change in GE performance and physiology, including the growth rate. Fish rely on external heat sources and suitable behaviors to maintain a constant body temperature in response to environmental changes. Numerous physiological activities and behaviors, including movement and digesting, are temperature-dependent, and fishes must continually prioritize their body temperature to meet these various requirements. Since the temperature sensitivity of different biological activities can vary, thermoregulation might result in possible conflicts between functions. To adapt to climatic changes such as global warming, understanding the importance of a fish species within the ecosystem requires knowledge of its dietary preferences and overall intake. An energy budget technique alone is insufficient for determining dietary choices. This can only be determined through a study of the gastric contents of fish collected in the wild. To turn quantifiable data on gastric contents into estimates of intake rates, it is required to understand the pace at which food items are eliminated from the stomach, that is, GER.

## 5. Conclusions

This study describes how temperature and type of diet significantly affected the GE of the adult hybrid Sabah grouper. These findings are important for improved management in the aquaculture of this newly developed hybrid grouper. Moreover, the modified square root model precisely fits the wet mass from GET, regardless of temperature and type of diet. The digestion results supported the growth indices (SGR, K) measured in hybrid adult Sabah grouper. The optimum temperature for the GE and growth was 30 °C. Faster GE and growth were observed in groupers fed with trash fish at all temperatures. Our findings can be used as baseline for future research on the hybrid Sabah grouper and as a model for managing and conserving similar species. The information obtained from this study will aid in improving the management of feed and maximizing production in grouper aquaculture.

## Figures and Tables

**Table 1:** Nutrient analysis (mean ± SE) of experimental feeds used.

Feed	Ash (%)	Moisture (%)	Protein (%)	Lipid (%)
Trash fish, *Sardinella* sp.	2.3 ± 0.15	78.5 ± 0.33	55.4 ± 0.62	7.3 ± 0.25
Pellet	11.4 ± 0.08	29.0 ± 0.01	37.5 ± 0.80	15 ± 0.13

**Table 2:** Summary statistics for parameter estimation of the square root model in stomach residuum for the adult hybrid Sabah grouper (the values are obtained from Figure 7).

Treatment	S_0_	*ρ*	*χ* ^2^	*r* ^2^
28 °C + pellet	29.26 ± 0.69	0.79 ± 0.02	2.24	0.98
30 °C + pellet	31.95 ± 1.44	0.57 ± 0.05	9.65	0.93
32 °C + pellet	24.90 ± 0.70	0.61 ± 0.02	2.43	0.97
34 °C + pellet	22.69 ± 0.63	0.95 ± 0.02	1.99	0.97
28 °C + trash fish	29.86 ± 0.72	0.52 ± 0.04	2.09	0.98
30 °C + trash fish	33.32 ± 1.61	0.41 ± 0.10	10.01	0.95
32 °C + trash fish	25.01 ± 1.48	0.45 ± 0.06	10.11	0.90
34 °C + trash fish	23.58 ± 1.32	0.55 ± 0.07	7.49	0.91

S_0_: average maximum meal size at time 0, *ρ*: gastric emptying rate, *χ*^2^: chi-square, and *r*^2^: regression co-efficient.

**Table 3:** Means of feeding rate, FCR, SGR, and K of hybrid Sabah grouper after 30 days of experimental period (mean ± SD).

Factor	Level	Feeding Rate (% day^−1^)	FCR	SGR (% day^−1^)	K
Temperature					
	28	1.30 ± 0.01 ^ab^	1.48 ± 0.06 ^ab^	8.33 ± 0.78 ^b^	1.28 ± 0.01 ^b^
	30	1.41 ± 0.02 ^a^	1.23 ± 0.12 ^c^	15.18 ± 1.07 ^a^	1.43 ± 0.02 ^a^
	32	1.23 ± 0.02 ^ab^	1.72 ± 0.06 ^ab^	4.39 ± 0.81 ^c^	1.23 ± 0.02 ^b^
	34	1.08 ± 0.03 ^c^	2.17 ± 0.32 ^a^	1.80 ± 0.69 ^d^	1.04 ± 0.03 ^c^
Diets	Pellet	1.24 ± 0.08 ^a^	1.80 ± 0.24 ^a^	6.59 ± 2.84 ^a^	1.22 ± 0.08 ^a^
	Trash fish	1.26 ± 0.07 ^a^	1.51 ± 0.16 ^a^	8.26 ± 2.99 ^b^	1.26 ± 0.07 ^b^
Diet × Temperature	28 °C + pellet	1.29 ± 0.02 ^a^	1.55 ± 0.10 ^a^	7.55 ± 1.61 ^a^	1.27 ± 0.01 ^a^
	30 °C + pellet	1.39 ± 0.04 ^a^	1.36 ± 0.02 ^b^	14.12 ± 1.05 ^b^	1.41 ± 0.03 ^b^
	32 °C + pellet	1.21 ± 0.02 ^a^	1.79 ± 0.02 ^c^	3.58 ± 1.74 ^c^	1.21 ± 0.03 ^c^
	34 °C + pellet	1.01 ± 0.06 ^a^	2.50 ± 0.65 ^d^	1.11 ± 1.42 ^d^	1.01 ± 0.04 ^d^
	28 °C + trash fish	1.31 ± 0.02 ^a^	1.42 ± 0.13 ^a^	9.12 ± 1.01 ^a^	1.29 ± 0.01 ^a^
	30 °C + trash fish	1.43 ± 0.01 ^a^	1.11 ± 0.25 ^b^	16.25 ± 2.11 ^b^	1.45 ± 0.04 ^b^
	32 °C + trash fish	1.26 ± 0.05 ^a^	1.66 ± 0.13 ^c^	5.20 ± 0.50 ^c^	1.26 ± 0.03 ^c^
	34 °C + trash fish	1.07 ± 0.03 ^a^	1.85 ± 0.05 ^d^	2.50 ± 1.55 ^d^	1.07 ± 0.05 ^d^

Different superscript letters (*p* < 0.05) indicate significant differences between each time points.

## Data Availability

Not applicable.

## Supplementary Materials

The following supporting information can be downloaded at: https://www.mdpi.com/article/10.3390/ani12223172/s1, Figure S1: Movement of pellet feed along the alimentary tract of the adult hybrid Sabah grouper by stomach dissection; Figure S2: Movement of trash fish feed along the alimentary tract of the adult hybrid Sabah grouper by stomach dissection.
