# Supplementary material for: Cross Effects of Diets and Rearing Temperatures on Gastrointestinal Evacuation and Growth Performance in Adult Sabah Groupers (Epinephelus fuscoguttatus × E. lanceolatus)
Source: Animals (Basel). 2022 Nov 16;12(22):3172. doi: 10.3390/ani12223172 (PMC9687046; doi:10.3390/ani12223172)
Supplement: Supplementary file 1 [file animals-12-03172-s001.zip › Supplementary S2-Das et al.pdf]

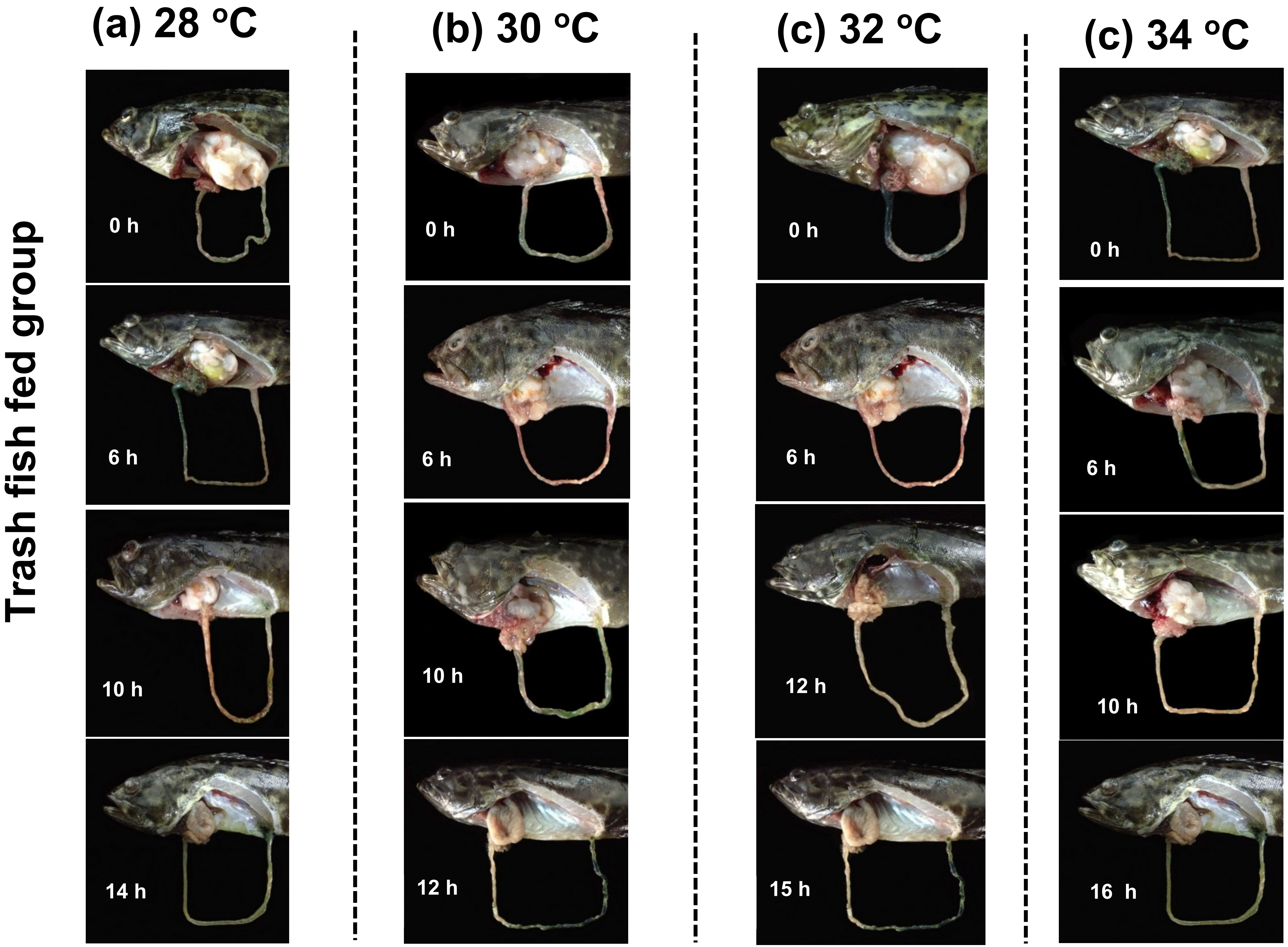

Figure S2. Movement of trash fish feed along the alimentary tract of the adult hybrid Sabah grouper by stomach dissection at (a) 28 °C, (b) 30 °C, (c) 32 °C, and (d) 34 °C. Five fish were used for each sampling point and temperature.
